# The Relative Concentrations of Nutrients and Toxins Dictate Feeding by a Vertebrate Browser, the Greater Glider *Petauroides volans*

**DOI:** 10.1371/journal.pone.0121584

**Published:** 2015-05-04

**Authors:** Lora M. Jensen, Ian R. Wallis, William J. Foley

**Affiliations:** Evolution, Ecology and Genetics, Research School of Biology, Australian National University, Canberra 0200, Australia; Portland State University, UNITED STATES

## Abstract

Although ecologists believe that vertebrate herbivores must select a diet that allows them to meet their nutritional requirements, while avoiding intoxication by plant secondary metabolites, this is remarkably difficult to show. A long series of field and laboratory experiments means that we have a good understanding of the factors that affect feeding by leaf-eating marsupials. This knowledge and the natural intraspecific variation in *Eucalyptus* chemistry allowed us to test the hypothesis that the feeding decisions of greater gliders (*Petauroides volans*) depend on the concentrations of available nitrogen (incorporating total nitrogen, dry matter digestibility and tannins) and of formylated phloroglucinol compounds (FPCs), potent antifeedants unique to *Eucalyptus*. We offered captive greater gliders foliage from two species of *Eucalyptus*, *E*. *viminalis *and *E*. *melliodora*, which vary naturally in their concentrations of available nitrogen and FPCs. We then measured the amount of foliage eaten by each glider and compared this with our laboratory analyses of foliar total nitrogen, available nitrogen and FPCs for each tree offered. The concentration of FPCs was the main factor that determined how much gliders ate of *E*. *viminalis* and *E*. *melliodora*, but in gliders fed *E*. *viminalis* the concentration of available nitrogen was also a significant influence. In other words, greater gliders ate *E*. *viminalis* leaves with a particular combination of FPCs and available nitrogen that maximised the nutritional gain but minimised their ingestion of toxins. In contrast, the concentration of total nitrogen was not correlated with feeding. This study is among the first to empirically show that browsing herbivores select a diet that balances the potential gain (available nutrients) and the potential costs (plant secondary chemicals) of eating leaves. The major implication of the study is that it is essential to identify the limiting nutrients and relevant toxins in a system in order to understand feeding behaviour.

## Introduction

Ecologists acknowledge that diet selection in browsing mammals involves some balance between the positive effects of nutrients and the negative effects of plant secondary metabolites (PSMs) [1,2]. It is, however, difficult to show that feeding on natural diets involves a trade-off between the acquisition of nutrients and the avoidance of toxins. This has prompted a plethora of studies over the past 30 years in which researchers attempt to relate the chemical composition of foliage to diet selection or the nutritional status of free ranging mammals e.g., [3–5], and yet, most of these studies prove inconclusive, sometimes for unknown reasons but often due to the difficulty in identifying the limiting nutrients and PSMs in the system, usually from a large number of intricately interacting possibilities. In lieu of this information, researchers often turn to the nutrients and PSMs that many regard as important. From the nutrient perspective, of most interest are often amino acids, measured as nitrogen (N) or crude protein, because there is a general consensus that they are the limiting nutrients for many herbivores [6,7]. This may be true as shown by Felton et al. [8], who found that meeting their protein requirements dictated how much spider monkeys ate. In contrast, Rothman et al. [9] found the opposite in mountain gorillas, which at times ingested more protein than they required in order to meet their energy requirements. This comparison emphasises the need to identify the limiting nutrients and the PSMs that either challenge detoxification systems or that reduce the digestibility of nutrients, rather than those believed to be important, such as total phenolics and fibre. Identifying the key factors for herbivore diet selection will prove invaluable as it will enable us to better understand their habitat choices, and, from a conservation point, conserve the correct habitats for herbivore species.

After a long series of studies, we have a good understanding of eucalypt chemistry and the factors that influence feeding on eucalypts by marsupial folivores e.g., [10–13]. Although species from the two most populous eucalypt subgenera, *Symphyomyrtus* and *Eucalyptus* (formerly *Monocalyptus*), commonly coexist [14] and attract different marsupial folivores [15] there are notable differences in their chemistry. Species of the subgenus *Symphyomyrtus* usually contain formylated phloroglucinol compounds (FPCs) [16] which do not occur in subgenus *Eucalyptus*. Leaves of species of the subgenus *Eucalyptus* typically contain about the same concentration of total N as do symphyomyrtles but only about half the concentration of available nitrogen (AvailN), due largely to the effects of tannins [17]. Tannins can reduce the digestion of nutrients in mammalian herbivores [18] largely via the formation of tannin-protein complexes, (depending on the specifics of tannin structure) and so can be a major influence on diet choice [19, 20]. If tannins make some proteins indigestible then concentrations of total nitrogen do not reflect the availability of protein to the herbivore. In contrast, available N (AvailN) measures the fraction of total N that is not bound to tannins or fibre [17,21] and thus is a more useful measure of the concentration of protein in a plant.

Apart from the substantial differences between the eucalypt subgenera, there is also large intraspecific variation in eucalypts in both the concentration of AvailN (but not total N) [22] and in FPCs [23] providing scope for studying trade-offs between nutritive factors and plant toxins known to be important. For example, studies of captive koalas (*Phascolarctos cinereus*), common ringtail possums (*Pseudocheirus peregrinus*) and common brushtail possums (*Trichosurus vulpecula*) indicate that FPCs are potent feeding deterrents [11,13,24,25]. Likewise, Moore and Foley [26] showed that wild koalas favour trees with lower concentrations of FPCs. In a different approach, DeGabriel et al. [27] studied a wild population of brushtail possums eating eucalypts of the subgenus *Symphyomyrtus* that surprisingly did not produce FPCs. They showed that females inhabiting home ranges with higher concentrations of AvailN (but not total N) had higher reproductive success and faster growing offspring than did females inhabiting poorer home ranges. In another field experiment, Youngentob et al. [28] found that greater gliders preferred monocalypts producing leaves with higher concentrations of digestible dry matter, total N and AvailN but preferred symphyomyrtles with lower concentrations of the FPC, sideroxylonal.

These data allowed us to test the hypothesis that food consumption depends on the balance between the potential gain (AvailN and digestible dry matter) and the potential costs (FPCs and tannins) associated with eating leaves. We chose the greater glider for this study because it feeds almost exclusively on young leaves of eucalypts with the preferred species often the dominant tall eucalypts in the habitat. Usually the diet consists of 3–6 different species of *Eucalyptus* over a year [29–32] but in some locations (e.g. SE NSW), a single species such as *E*. *viminalis* can comprise a large part of the diet year-round. Thus in captivity we provided 4–6 species for general maintenance of the species but focussed our studies on the single species. *E*. *viminalis* and *E*. *melliodora* because we have a good understanding of chemical variation of both species and in the case of *E*. *melliodora* we aimed to make comparisons with other folivores [25]. Another advantage of *E*. *melliodora* is that individuals vary widely in their production of sideroxylonals which allowed us to offer foliage with a wide range of FPC:AvailN concentrations.

## Methods

### Capture and housing

Four female and four male greater gliders (*Petauroides volans*) (mean body mass 1096 g; sd 195 g) were captured in Bungongo State Forest, Tumut, NSW (35° 02' S 148° 28' E) in March 2009—the non-breeding season. We used a spotlight to locate animals on exposed branches and a 0.222 calibre rifle to break the branch on which the animal perched. We captured the animal as it glided to the ground, placed it in an open weave sack and transported it to the animal house facilities at the Australian National University, Canberra, Australia. This research was approved by the Animal Experimentation Ethics Committee of the Australian National University (Permit number F.BTZ.33.08) and conforms to the Australian Code of Practice for the Care and Use of Animals for Scientific Purposes. This research was conducted with the permission of State Forests New South Wales (Permit XX43632) and NSW National Parks and Wildlife Service (Permit S12812).

The animals were held in individual metabolism cages (60 x 80 x 60 cm) as described by Foley and Hume [33]. The cages were in a room maintained at 20 ± 3°C under a 12h:12h light:dark cycle with a gradual change in intensity over 30 min to simulate dawn and dusk. Each cage contained a T-shaped timber perch, a water source and a wooden nest box. Stems (ca 40 cm and 350 g) of *Eucalyptus* foliage were placed in a tube of water outside the cage with the foliage protruding inside. Animals were weighed at the beginning and end of each experimental period.

### Diet and feeding

When first caught and when not being used in experiments, gliders were maintained on a mixed diet of *Eucalyptus radiata* and *E*. *dives* (sub-genus *Eucalyptus*), and *E*. *bridgesiana*, *E*. *melliodora*, *E*. *viminalis*, and *E*. *grandis* (sub-genus *Symphyomyrtus*) offered *ad libitum*. We collected branches of experimental foliage from individually identified *E*. *melliodora* (N = 16) and *E*. *viminalis* (N = 15) trees, whose chemistry we knew, at sites within 50 km of Canberra and stored them for up to 10 d by placing them in large polythene bags with the ends of branches in tubs of water in a cool-room (4° C).

The daily routine remained the same in all feeding experiments. We prepared bunches of foliage at 14:00 h and left them to equilibrate in water before re-weighing and placing them in cages at 16:00 h. Buds or fruits were removed to ensure measurements of intake were for foliage only. Changes in the dry matter content of the foliage were assessed with control bunches of foliage kept in the same room as the animals. Uneaten foliage was removed from the cages and weighed at 09:30 h, while spilled foliage was collected and dried to constant mass at 40° C for 48 h. This information enabled us to determine how much the gliders ate, expressed as dry matter intake (DMI).

### Design of feeding experiments

We measured feeding by greater gliders in response to FPCs and AvailN using *E*. *viminalis* and *E*. *melliodora*. We selected trees based on their concentration of FPCs and AvailN to give a wide range of FPC:AvailN values. *E*. *viminalis* contains several FPCs, including sideroxylonals and various macrocarpals [23]. In contrast, sideroxylonals are the only FPCs produced by *E*. *melliodora* so this species is ideal for testing the hypothesis proposed by Youngentob et al. [28] that sideroxylonals rather than other FPCs determine the trees that greater gliders visit.

We chose to use single-choice protocols to increase our statistical power to detect the role of FPCs and AvailN in feeding choices. We used cyclical changeover designs that varied depending on the number of animals available but in all cases the animals were offered the leaves of a treatment tree for one night and thus could choose only how much they ate as opposed to selecting individual trees or species. In the first study, with *E*. *viminalis*, we fed 15 trees to seven or eight animals with four or five measures of food intake per tree. In the experiment with *E*. *melliodora*, we fed the leaves of 16 trees to eight gliders giving four measures of food intake for each tree. Each eucalypt species was offered to the gliders over the course of two experimental periods in which experimental nights alternated with rest nights and a rest period of one week separated the experimental blocks. On these rest nights and during the rest periods, we offered the gliders a mixture of foliage from several species described previously under “Diet and feeding”.

### Diet analysis

Two leaf samples representing the foliage eaten by the gliders were taken from each control bunch and placed in paper bags. We placed one sample, weighing approximately 10 ± 1 g wet matter, in a 40° C oven for 48 h to calculate DM while the other sample, destined for analysis, was stored at -20° C before being freeze-dried and ground to pass a 1 mm screen in a Tecator cyclone mill.

### Formylated phloroglucinol compounds

We extracted and quantified sideroxylonals (the only FPCs in *E*. *melliodora*) following the method of Wallis and Foley [34], which uses sonication followed by isocratic HPLC separation (93% acetonitrile, 7% water, both with 0.1% trifluoroacetic acid) lasting 12 min. We used the same method to extract FPCs from *E*. *viminalis* but because this species contains several FPCs, we used an 80 min HPLC procedure to separate and analyse the FPC compounds Extracts were eluted under gradient conditions with 0.1% TFA in acetonitrile (A) and 0.1% TFA in water (B) as follows: 60% A/40% B for 5 min, linear gradient to 90% A/10% B at 60 min, hold for 10 min and return to starting conditions by 80 min. We used a Waters Alliance Model HPLC fitted with photo diode array detector for all separations. The analytical column was a Wakosil 250 × 4 mm GL 3C18RS (SGE) held at 37° C and the flow rate was 0.75 ml min^-1^. We measured the peak response at 275 nm.

### Available nitrogen

**Fig 1:**
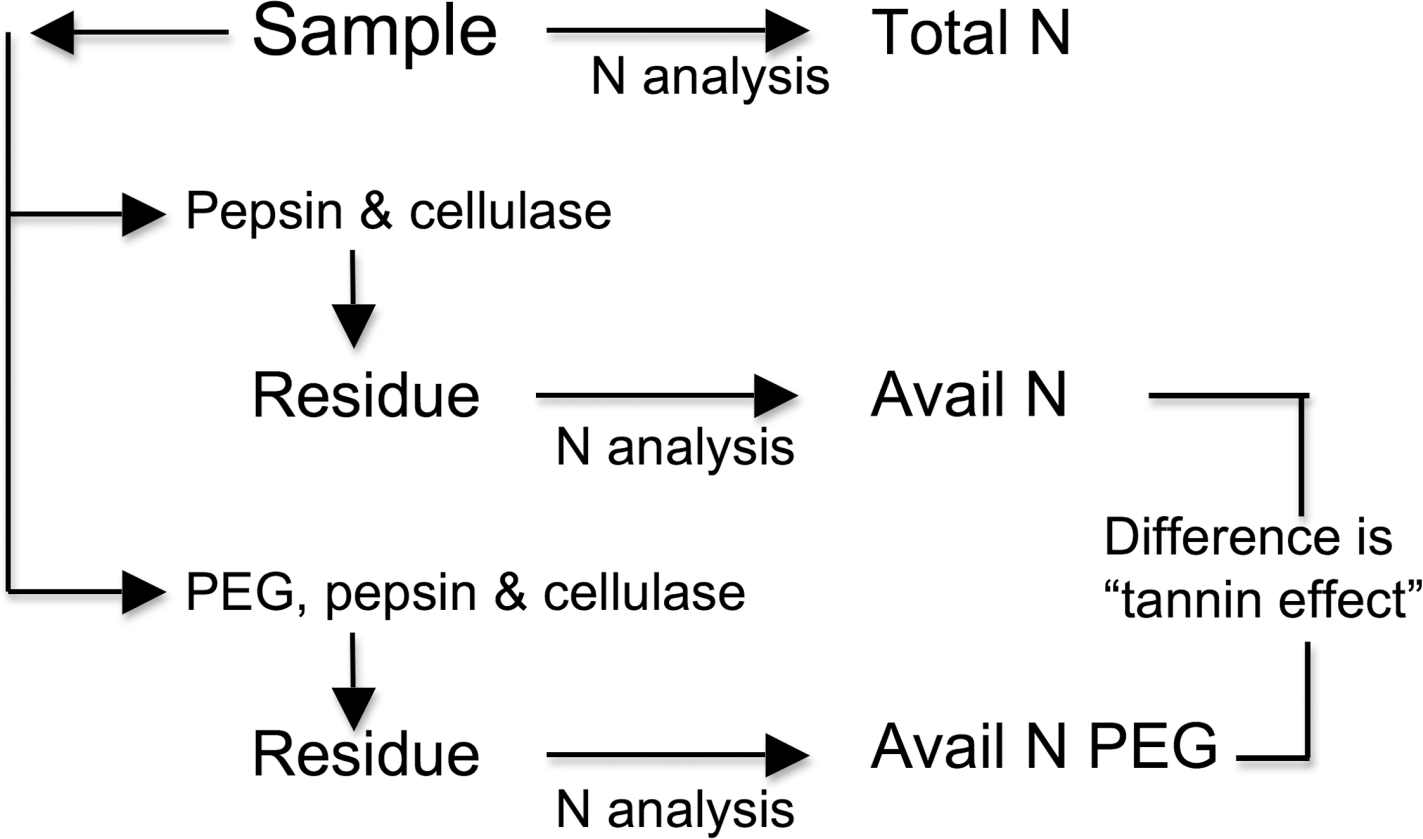
Schematic diagram showing the relationship between the Total N, Available N and Available N PEG as measured *in vitro*. The difference between the concentration of Available N and Available N PEG, is an estimate of the effect of tannins complexing with proteins in the sample.

We determined *in vitro* AvailN of all leaf samples using the method of DeGabriel et al. [22] as modified by Wallis et al. [17] (Fig 1). Briefly, we incubated 800 ± 10 mg samples in ANKOM F57 filter bags (Macedon, U.S.A.) in a series of buffers and enzymes, at 35° C with constant stirring. At the start, we incubated two bags per sample in 0.05M Tris-Base buffer (for AvailN) and another two bags in buffer containing 33.3 g.L^-1^ of polyethylene glycol (PEG) 4000 (for AvailNPEG). PEG can disrupt tannin-protein complexes and thus its inclusion in the *in vitro* procedure allows an estimate of the extent to which tannins are limiting *in vitro* dry matter and N digestibility. After 24 h we washed the samples before drying them to constant mass and reweighing them. We then digested the samples in pepsin (2 g.L^-1^ 1:10 000 pepsin in 0.1N HCl) for 24 h, followed by cellulase (6.25 g.L^-1^ cellulase in acetate buffer, pH 4.8) for 48 h. Finally, after thorough washing, they were dried to a constant mass at 50° C, weighed and the contents were analysed for N. All N analyses were done on a Leco Truspec Carbon/Nitrogen Determinator (Leco, St. Joseph, U.S.A.) using 200 ± 10 mg of leaf sample and 100 ± 10 mg of sample from the *in vitro* digestion bags. From this, we calculated *in vitro* dry matter digestibility and N digestibility (%) as the proportional losses of dry matter and N from the original sample respectively, while AvailN (% DM) is the product of the concentration of total N and *in vitro* N digestibility. The difference in available N between samples incubated with (AvailNPEG) and without PEG (AvailN) is estimated as the effect of tannins [17,21] (Fig 1).

### Statistical Analysis

We analysed the data in GenStat Release 10.2, Lawes Agricultural Trust (Rothamsted Experimental Station). First we examined relationships between the chemical measures of foliage using simple linear regression. We then used the restricted maximum-likelihood algorithm (REML) to analyse the factors that influenced feeding by gliders. For gliders fed both *E*. *viminalis* and *E*. *melliodora*, we tested the following model:

Response Dry matter intake

Fixed model: Constant + Period + AvailN + FPC + AvailN*FPC + Body Mass

Random model: Glider + Period + Glider*Period

“Period” refers to the two experimental periods that were part of each feeding study (see experimental design), while AvailN and FPC refers to the concentration of these entities in the leaves we fed. We progressively dropped non-significant terms (P>0.1) from the model leaving a reduced model on which we based the results.

We tested the normality of the residual plots after developing each model and the second order Akaike information coefficient (AICc) to guide selection of the models to ensure, that dropping a term resulted in a better model. The body mass of the gliders did not affect how much they ate so we present results of DMI as g per animal per day.

## Results

### Variation in the concentrations of AvailN and FPCs in leaves from experimental trees

Leaves from individual *E*. *melliodora* and *E*. *viminalis* varied widely in their concentrations of FPCs (coefficient of variation, CV = 39% and 36%, respectively) and AvailN (CV = 23% and 26%) but there was less variation in the concentration of total N (CV = 13% and 10%). There was even less variation in *in vitro* dry matter digestibility (CV = 6% and 6%) (Table 1) with a seven-fold range in the ratio of the concentrations of FPCs to AvailN in *E*. *viminalis* and an eleven-fold range in *E*. *melliodora* (Table 1).

**Table 1:** Concentrations of nitrogen and formylated phloroglucinol compounds in the leaves of *Eucalyptus melliodora* (N = 16) and *Eucalyptus viminalis* (N = 15) fed to greater gliders.

Species	Statistics	Sideroxylonal (mg.g^-1^ DM)	Other FPCs (mg.g^-1^ DM)	Total N (% DM)	AvailN^1^ (% DM)	AvailNPEG^2^(% DM)	DMD ^3^ (%)	FPC: AvailN ^4^
*E*. *melliodora*								
	Mean	29.6	0	1.53	0.84	1.02	64.4	38.0
	SD	11.4	0	0.20	0.20	0.21	4.1	22.4
	CV % ^5^	39	0	13	23	21	6	59
	Minimum	6.2	0	1.23	0.44	0.71	58.3	7.5
	Maximum	55.7	0	1.92	1.24	1.53	70.1	84.6
*E*. *viminalis*								
	Mean	3.7	15.6	1.50	0.85	1.13	54.2	25.3
	SD	5.5	6.0	0.16	0.22	0.18	3.0	13.1
	CV %	151	36	10	26	16	6	51
	Minimum	0.6	7.0	1.24	0.60	0.83	48.8	6.9
	Maximum	22.8	23.7	1.76	1.32	1.45	59.1	48.6

^1, 2^ Available N and available N in the presence of PEG measured in an *in vitro* assay

^3^ Dry matter digestibility measured *in vitro* assay

^4^ The ratio of the concentrations of FPC and available N shown to indicate the range of values

^5^ Coefficient of variation

The concentration of AvailN was related to the concentration of total N in both *E*. *melliodora* (AvailN = -0.27 + 0.73 x Total N; r^2^ = 0.56; F _1,14_ = 18.0; P < 0.001) and in *E*. *viminalis* (AvailN = -0.63 +0.99 x Total N; r^2^ = 0.50; F_1,13_ = 13.2; P = 0.003). The concentration of AvailNPEG was also related to that of total N in both species. The relationship, however, compared to that for AvailN was weaker in *E*. *melliodora* (AvailNPEG = -0.07 + 0.71 x Total N; r^2^ = 0.44; F _1,14_ = 11.4; P = 0.004) but stronger in *E*. *viminalis* (AvailNPEG = -0.38 + 1.00 x Total N; r^2^ = 0.72; F _1,14_ = 34.4; P < 0.001).

We found no relationship between the concentrations of FPCs and N or AvailN in either *E*. *melliodora* (P = 0.99, P = 0.29) or in *E*. *viminalis* (P = 0.92, P = 0.46) and between FPCs and AvailNPEG in *E*. *viminalis* (P = 0.38). There was, however, a relationship between FPCs and AvailNPEG in *E*. *melliodora* (FPC = 61.3–30.9 x AvailNPEG; r^2^ = 0.34; F _1,14_ = 7.2; P = 0.017).

### Feeding by greater gliders in response to plant chemistry

Greater gliders willingly ate leaves from both species of eucalypt and gained a little weight (mean = 0.79%; se = 1.42) during the *E*. *viminalis* experiments but lost weight (mean = -5.16%; se = 0.91) while eating *E*. *melliodora*. The body mass of the gliders had no effect on how much they ate (*E*. *melliodora* P = 0.49; *E*. *viminalis* P = 0.90; REML).

Even though they ate roughly similar amounts of the two species, the gliders responded differently to the plant chemistry, with the concentration of sideroxylonals being the only significant (P = 0.007) attribute of plant chemistry that influenced feeding in gliders offered *E*. *melliodora*. Total N (P = 0.25), AvailN (P = 0.60), AvailNPEG (0.76) and dry matter digestibility (P = 0.50) all had no bearing on how much the animals ate (see to Eq 1).

**Fig 2:**
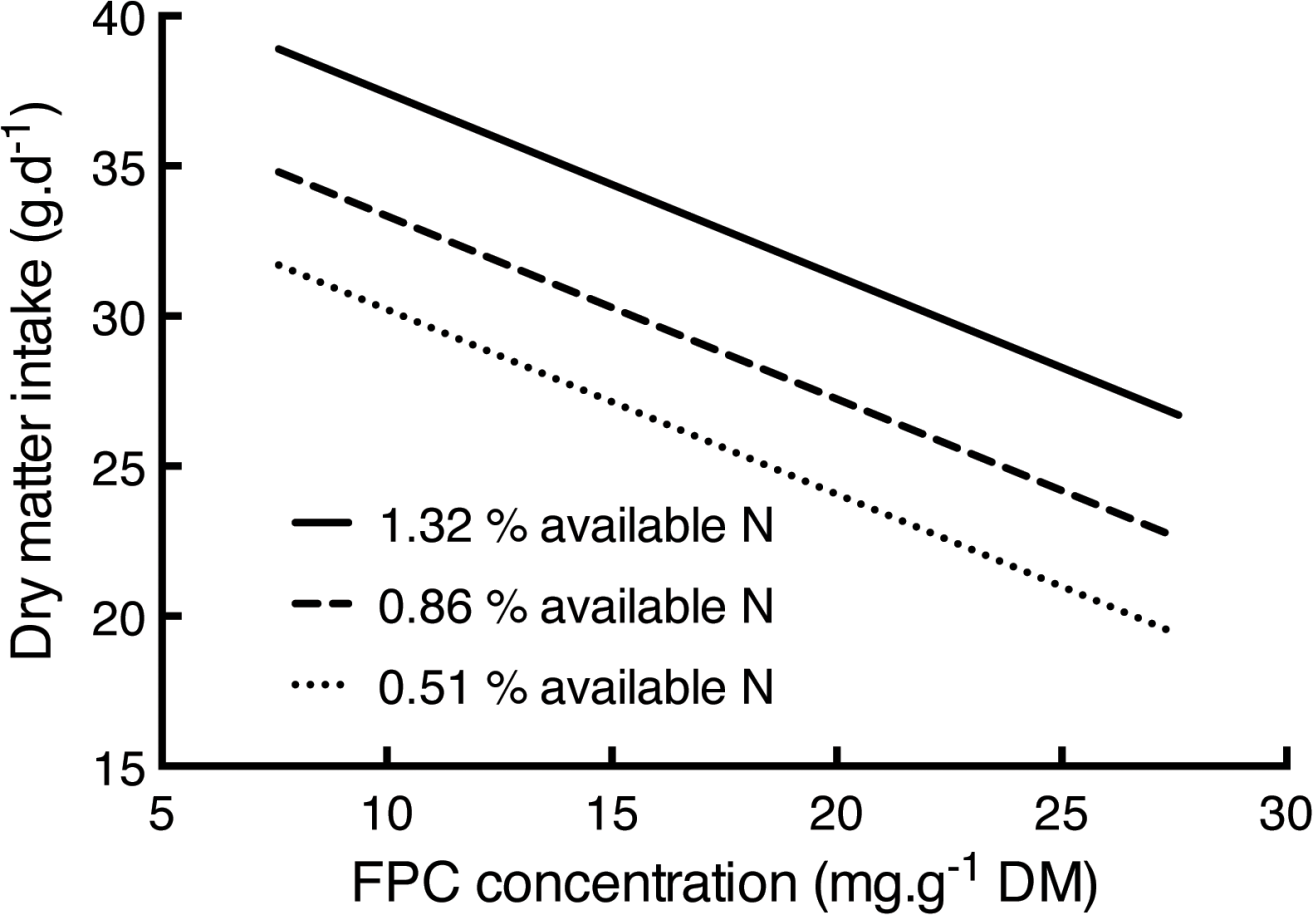
The predicted amounts of *E*. *viminalis* eaten by greater gliders in relation to observed concentrations of formylated phloroglucinol compounds. The lines represent predicted dry matter intakes at three concentrations of AvailN. Minimum observed (small dashed line; 0.51% DM), mean (larger dashed line; 0.86% DM) and maximum (solid line; 1.32% DM). Confidence intervals were omitted to simplify the graph but errors around the measures of the constant, AvailN and FPCs appear in Eq 2.

In contrast, the amount of *E*. *viminalis* that gliders ate depended on the concentrations of AvailN (P = 0.018) and of FPCs (P < 0.001) (Fig 2). The model from REML indicated positive effects on food intake for AvailN countered by the negative response to total FPCs (see Eq 2 for the model with the covariates centred (mean AvailN = 0.859; mean FPC = 17.55). Furthermore, separating the FPCs into sideroxylonals and non-sideroxylonal FPCs did not improve the model indicating that greater gliders respond similarly to all FPCs (ΔAICc = 0.85). Also, *in vitro* dry matter digestibility, which ranged from 49 to 59% did not influence food intake (P = 0.45).

$$DMI\left(g.d^{-1}\right)=46.83\;\left(se\;4.24\right)-0.37\;\left(se\;0.11\right)\times Sideroxylonal\left(mg.g^{-1}DM\right)$$ Eq 1

$$DMI\left(g.d^{-1}\right)=28.83\;\left(se\;0.61\right)+8.84\;\left(se\;3.75\right)\times AvailN-0.608\;\left(se\;0.12\right)\times FPC\left(mg.g^{-1}DM\right)$$ Eq 2

## Discussion

In this study, we confirmed our first hypothesis that the amount of *E*. *viminalis* foliage that greater gliders ate depended on the balance between the potential gain of nutrients (AvailN) and the potential costs (FPCs) associated with eating leaves. This seems intuitive but demonstrating the point required a long and arduous series of experiments on arboreal folivores with two key breakthroughs that identified explanatory traits. The first of these was the discovery of the primary feeding deterrent, the FPCs, in *Eucalyptus ovata* [12] and subsequent survey that showed they occur in species of the subgenus *Symphyomyrtus* but not in the subgenus *Eucalyptus* [16]. A series of feeding experiments with captive animals demonstrated the potency of these compounds [25], while a field survey indicated their importance in tree-selection by several species [13,26]. The second breakthrough was tackling the role of tannins from a functional angle, which allowed the protein-binding capacity of tannins to be incorporated into the measure of AvailN [17,21].

Expressed differently, a key finding of this study was that total N had no bearing on food intake because the total N in the leaves we fed the gliders did not reflect its availability largely due to the influence of tannins. The ubiquity of tannins means that the findings extend much further than marsupials eating eucalypt leaves; they are relevant to any vertebrate herbivore eating tannin-rich material. Thus, in these cases, we see no value in using total N or broad measures of tannins, such as total phenolics, as explanatory variables and instead urge researchers to measure what is important to the animal.

A second advantage of the AvailN assay is that it accounts for the N-binding effects of tannins and fibre and so precludes the need to concoct ratios, such as total N:fibre or total N:total phenolics to weight the good and the bad attributes of a food. Such ratios, often of traits that researchers think are important, are commonplace in ecology. Frequently this approach is inconclusive e.g., [35], while in other cases it seems to work. For instance, Cork and Catling [36] predicted the abundance of arboreal folivores in eucalypt forests from the concentrations of N and total phenolics. These relationships, however, are simply correlative and without further experimentation to identify explanatory traits may merely indicate some unmeasured factor. The nitrogen-to-fibre ratio proposed by Milton [37] to explain leaf selection by primates and since used to predict the biomass of folivorous monkeys at local [38,39] and regional [40–43] scales is another example. Wallis et al. [21] suggested that this ratio might give the “right answer for the wrong reason” because the concentration of total N in plants often does not reflect its availability to animals. This critique garnered support from Gogarten et al. [44] who did not find higher densities of red colobus monkeys in regenerating forests where leaves had more favourable ratios of protein to fibre. Another risk that ratios pose is that they make something appear significant when in fact it is not. In the current study, the ratio of FPCs to total N had a significant influence on the quantity of *E*. *viminalis* leaves that greater gliders ate (F_1,62_ = 25.6; P < 0.001), but total N had no effect when included in a model with FPCs (F_1,56_ = 0.05; P = 0.82)

In contrast, AvailN influenced the amount gliders ate and while the effect may appear subtle, relative to that of the FPCs, DeGabriel and colleagues [27] showed that common brushtail possums (*Trichosurus vulpecula*) inhabiting home ranges where the trees produced leaves with lower mean concentrations of AvailN (0.30% DM) had lower reproductive success and slower growing offspring than did possums occupying better home ranges (AvailN = 0.40% DM). Similarly, McArt et al. [45] reported lower fecundity and twinning rates in moose where the AvailN concentration in the summer food was lower. In both of these studies, tannins were mainly responsible for limiting the availability of N and our data supports those findings. Furthermore, Felton et al. [8] showed that spider monkeys tightly regulate their intake of AvailN but not of total N.

The apparently subtle effect of AvailN is the likely explanation for the different responses of gliders to *E*. *melliodora* and *E*. *viminalis*. Both eucalypt species have similar mean values of AvailN but the higher concentrations of AvailNPEG (*E*. *viminalis* = 1.13% DM; *E*. *melliodora* = 1.02% DM) indicate that tannins are more active in binding protein in *E*. *viminalis*. This variation in *E*. *viminalis* provides the scope for gliders eating the species to select their diet using both FPCs and AvailN.

Our study identifies the tenuous balance between nutrients and plant secondary compounds in one system—the greater glider consuming eucalypt foliage. Nevertheless our findings have much wider implications. Ecologists wanting to explain the uneven distribution of animals across landscapes often seek nutritional explanations. Identifying nutritional factors that predict abundance, however, has proven remarkably difficult. While most researchers agree that the nutritional value of a landscape depends on both positive (concentrations of available nutrients) and negative (concentration of plant defences) traits, identifying these traits and understanding their interactions is much harder. This study confirms this thesis, shows the importance of identifying the crucial factors, in this case AvailN and FPCs and confirms what we might expect and what others report—that, due to secondary compounds, there may be a very fine balance between nutrient gain and nutrient loss.
